# Decoding the mysteries of prostate cancer via cutting-edge liquid biopsy-based urine exosomes profiling

**DOI:** 10.1016/j.jlb.2024.100162

**Published:** 2024-07-14

**Authors:** Swarup Sonar

**Affiliations:** Center for Global Health Research, Saveetha Medical College and Hospital, Saveetha Institute of Medical and Technical Sciences, Saveetha University, Chennai, 602105, Tamil Nadu, India

**Keywords:** Exosomes, Prostate cancer, Urine exosomes, AI, Sensor, Liquid biopsy

## Abstract

Exosomes, a subset of extracellular vesicles (EVs), have emerged as crucial players in prostate cancer (PCa) diagnosis. It is involved in cell-to-cell communication. Exosomes cargo based tumor microenvironment (TME) cellular communication is a secret of cancer development and progression. PCa, the second most common cancer in men, holds early diagnostic challenges due to a lack of specific biomarkers. Recent studies have found that tumor-derived exosomes (TEXs) contribute to PCa progression by facilitating immune suppression, angiogenesis, and metastasis. These exosomes, detectable in serum, plasma, and urine, carry diagnostic biomarkers such as specific miRNAs and proteins. Urine-derived exosomes, with their distinct miRNAs, provide a non-invasive diagnostic tool. This method becomes cutting-edge liquid biopsy in PCa. Advanced techniques, such as including AI and nanoplatform-based sensors, improve the precision of exosome-based PCa diagnostics, offering enhanced detection and better treatment outcomes.

Cancer is one of the most significant global health challenges. Among these, prostate cancer (PCa) is a prevalent and serious threat to men worldwide, it is the second most frequent cancer in males [1]. PCa early diagnosis is very challenging due to lake of specific biomarkers (prostate-specific antigen-PSA is a widely used biomarker for PCa but is not an accurate result). Extracellular vesicles (EVs) introduce a new chapter in cancer biology. EVs are secreted from active cells and they acts as signalling molecules in cell-to-cell communication. The most exciting fact cells derived EVs nature depict parental cells. This concept clicks in the scientific mind and investigation begins EVs based cell nature prediction. The investigation found that during cancer development, tumor microenvironment (TME) released extracellular vesicles (EVs) play a vital role in tumor cellular communication [2]. In general, EVs are classified into three major subpopulations such as microvesicles, apoptotic bodies and exosomes. In current decade exosomes are most highlighted in cancer research [3]. In TME exosomes biogenesis, depend on the hypoxic condition and P^H^ of TME. Exosomes biogenesis (in cancer) is regulated via the ESCRT complex independent pathways. Tumor derived exosomes (TEXs) reprogram cell populations in TME and promote cancer (Fig. 1). In PCa, TEXs regulate several stages of cancer such as uncontrolled cell growth, immune suppression, angiogenesis, metastasis, and organ-specific metastasis.

**Fig. 1:**
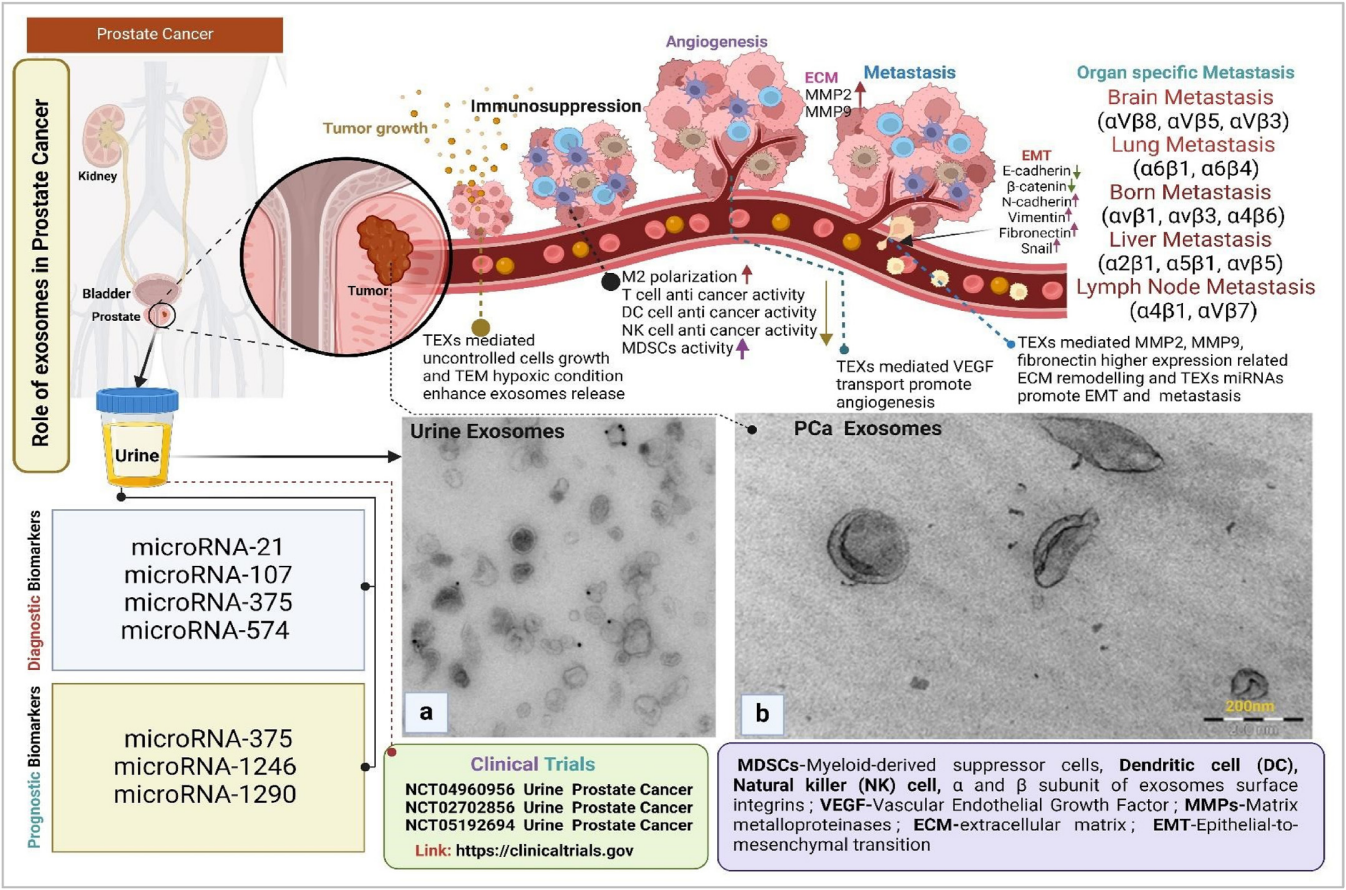
**Clinical impact of urine exosomes in prostate cancer. a)** Urine-derived exosomes (Reproduced with permission under Creative Commons CC BY 4.0 license from ref.[24]. Copyright @ 2015 The Authors), **b)** Prostate cancer exosomes (Reproduced with permission under Creative Commons CC BY 4.0 license from ref.[25] Copyright @ 2018 The Authors).

In prostate cancer, TME-assisted hypoxia TEXs promote cancer (PCa) progression [4]. Immune suppression during the progress of cancer immune cells promotes cancer because of their altered functional phenomena [5]. PCa cells derive exosome-mediated IL-8 transported reprogram T-cell metabolism and develop immune suppression [6]. Cancer-altered metabolic activity requires more amount of nutrients, via the angiogenesis process new blood vessels formation fulfils this requirement (in general TEXs mediated VEGF promoter angiogenesis) [7]. In PCa Exosomal PGAM1 promotes angiogenesis and metastasis [8]. Exosome-mediated miRNA-9 plays a vital role in PCa metastasis [9]. It may be a therapeutic target for PCa. In cancer, circulated tumor cells migrate in the secondary tumor site via the guidance of exosome surface integrin [10]. Exosomes also vital messengers of PCa. Exosomes cargos-based PCa biomarkers are very significant. Exosomes derived from urine as a source of biomarker, enable early and precise non-invasive diagnosis of cancer, particularly prostate cancer, offering a promising approach for improving detection and treatment outcomes. Exosomes derived from serum, plasma, and urine play pivotal roles in prostate cancer (PCa) pathogenesis through their distinct cargo compositions and functional roles. Serum-derived exosomes are characterized by miRNA-375 and miRNA-141, serving as diagnostic markers while potentially enhancing tumor progression [11]. Plasma-derived exosomes enriched with miRNA-1290 and miRNA-375 function as prognostic markers, indicating their involvement in castration-resistant PCa (CRPC) progression [12]. Exosomes derived from plasma and serum in prostate cancer (PCa) feature αvβ3 integrin proteins, serving as dual prognostic and diagnostic markers that promote an aggressive PCa phenotype [13]. Urine analysis gets priority in cancer due to some facts such as non-invasive (it is easy to collect compared to blood, CSF etc.), enrichment of cancer markers (DNA, RNA, proteins etc.), easy of monitoring (use of spectroscopy, mass spectroscopy and next generation sequencing easy to detect cancer biomarkers), potential for point of care testing (this method is cost-effective), and specific for prostate cancer. The presence of known PCa biomarkers in urine-derived exosomes indicates that exosomes become a source of diagnostic biomarkers for PCa [14]. Urine exosomes-based dynamic cargo molecules such as ERG, PCA3, and SPDEF mRNAs, promote an aggressive PCa phenotype [15], alongside a spectrum of miRNAs like miRNA-196, miRNA-501, miRNA-34, miRNA-92, miRNA-143-3p (downregulated) [16] and miR-574-3p, miR-141-5p, miR-21-5p (upregulated) [17], crucial for PCa diagnosis. Moreover, urine-derived exosomes also feature lncRNAs such as lncRNA-21, which acts as a diagnostic marker [18]. Urine-based PCa biomarker investigation is currently getting huge priority in cancer research [19]. AI and machine learning develop smart platforms for more precise biomarker detection and early diagnosis of PCa (Fig. 2). In this process, collective data is analysed via ML algorithm for biomarker scanning and within a short time biomarkers are detected [1]. A combination of AI and exosome biology develops a precision, effective and affordable solution-based digital health system for PCa.

**Fig. 2:**
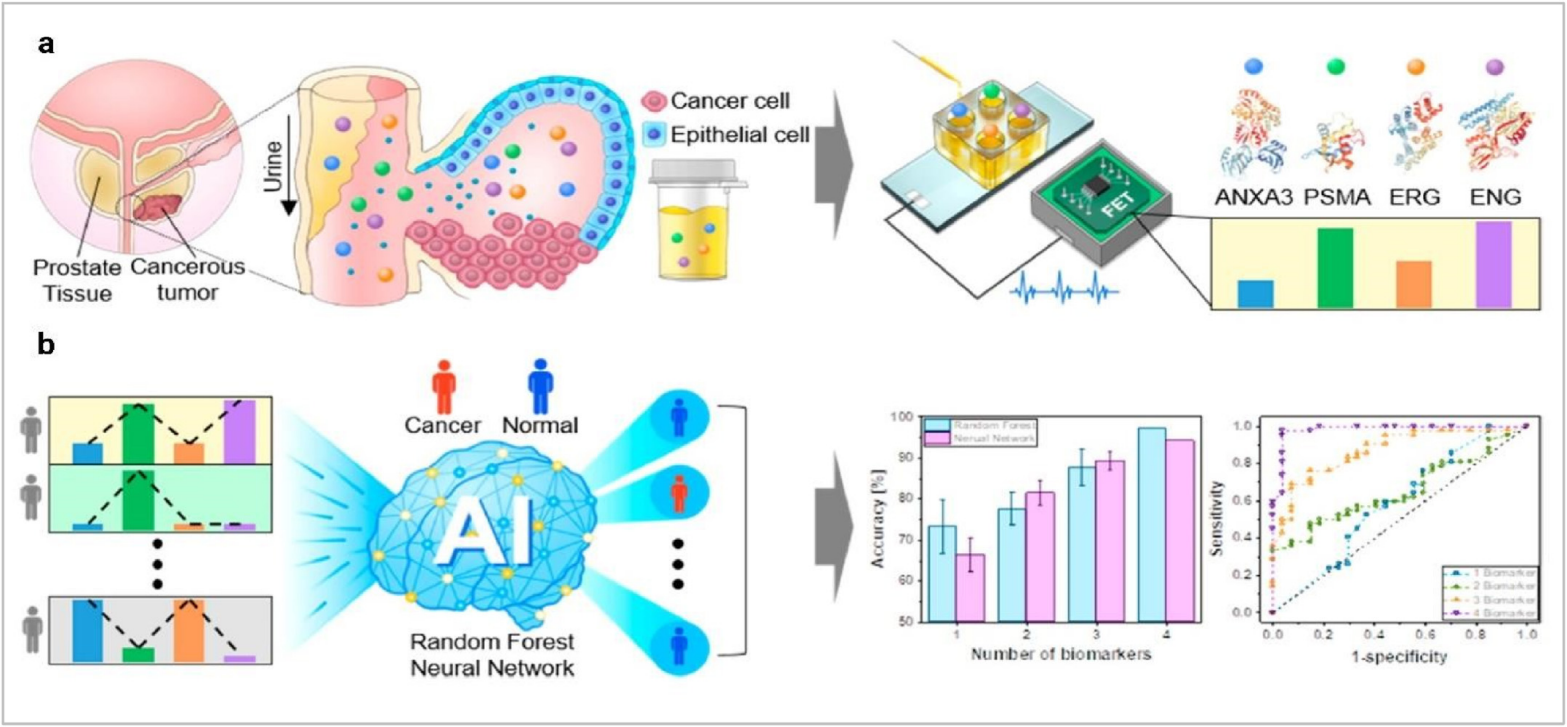
**Urine exosomes analysis via AI. a)** measures the electrical signals from the four different biomarkers in the urine, **b)** The set of sensing signals collected for each patient were then analysed using ML to screen the patient for PCa. RF and NN algorithms to analyse the multimarker signals. Both algorithms provided an increased accuracy, and the AUROC increased in size as the number of biomarkers was increased (Reproduced with permission from ref. 1 Copyright @ 2021 American Chemical Society.).

Patient serum exosomes present p-glycoprotein, a diagnostic marker of docetaxel resistance in PCa [20]. Current decade non-invasive spectroscopy-based PCa exosome profiling become more highlighted [21]. Nanoplatform-based exosome sensors and advanced methods create a landmark of precision PCa diagnostic era with effective high performance (Fig. 3).

**Fig. 3:**
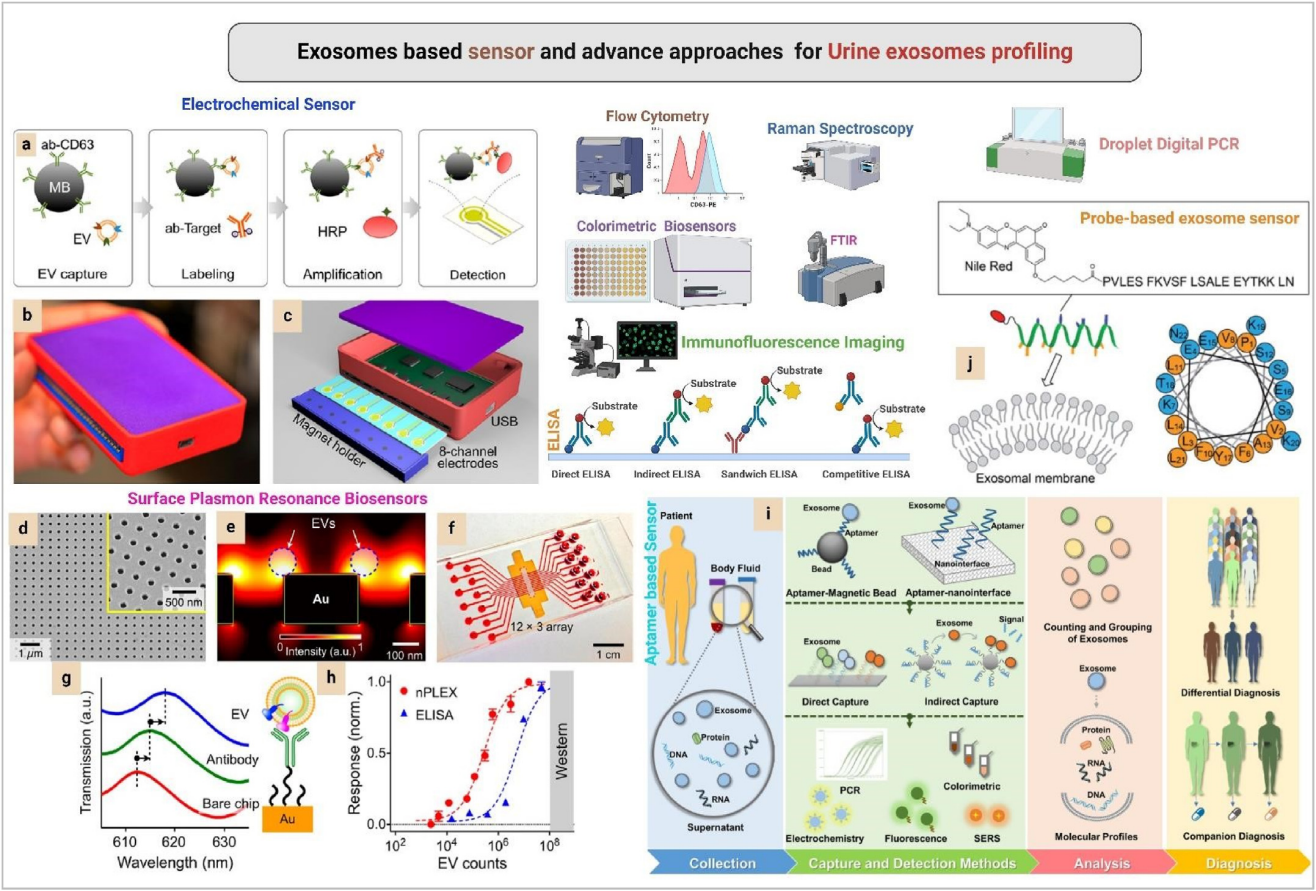
**Advanced approaches for urine exosomes analysis. a)** Exosomes-based electrochemical sensor working Stapes, **b)** A image of Integrated Magnetic-Electrochemical Exosome (iMEX) Platform, **c)** different parts of iMEX, **d)** The nPLEX (Nanoplasmonic Exosome) sensing is based on transmission SPR (surface plasmon resonance) through periodic nanohole arrays, **e)** Finite-difference time-domain simulation shows the enhanced electromagnetic fields tightly confined near a periodic nanohole surface. The field distribution overlaps with the size of EVs captured onto the sensing surface, maximizing the detection sensitivity, **f)** The sensing array can be integrated with multichannel microfluidics for independent and parallel analyses, **g)** Assay schematic of changes in transmission spectra showing EV detection. The gold surface is prefunctionalized by a layer of polyethylene glycol (PEG), and antibody conjugation and specific EV binding were monitored by transmission spectral shifts as measured by sensor, **h)** In comparison to gold standard methods, the nPLEX assay demonstrated excellent sensitivity, it more sensitive than Western blotting and chemiluminescence ELISA, respectively. (Fig. 3. a to h Reproduced with permission from Ref. [26] Copyright @ 2018 American Chemical Society.), **i)** Aptamer-based exosome sensor (Reproduced with permission from Ref. [27] Copyright @ 2021 American Chemical Society.), **j)** Probe exosome sensor (Reproduced with permission under Creative Commons CC BY 4.0 license from Ref. [29] Copyright @ 2020 RSC Adv.). (For interpretation of the references to colour in this figure legend, the reader is referred to the Web version of this article.)

Advanced approches such as electrochemical sensor (this approach is more sensitive compared to ELSA and Western blot), surface plasma resonance sensor (this process highly sensitive and highly specific for tumor exosomes scaring), flow cytometry, colourimetric sensor, Immunofluorescence image, ELISA, aptamer-based sensor, and digital droplet PCR (this method allows exosomes carry muted DNA fragment analysis) supports precise urine exosomes profiling. Urine exosome-based PCa profiling (this method is a combination of exosome biology, nanotechnology, AI and ML) supports precision early PCa detection. In future, this interdisciplinary approach become a promising smart platform upcoming digital health care. Worldwide ongoing exosomes-based clinical trials [28], [29], indicate exosomes get huge priority in cancer research at current time. The limitations of the clinical application of exosomes are heterogeneity (exosomes origin, size, molecular variation) [30], lack of standard isolation method, and storage of exosomes. Urine exosome-based single exosome profiling, exosome barcoding, muti-omics approach, and AI-based exosome profiling become a remarkable phenomenon for exosome-based cancer biomarker investigation [22,23]. Exosome becomes a rising star of transforming precision cancer diagnostic era.

## Availability of data and materials

Data sharing is not applicable to this article as no datasets were generated or analysed during the current study.

## Funding

There is no funding for this study.

## Ethical approval/patient consent

Not Applicable.

## Declaration of competing interest

The authors declare that they have no known competing financial interests or personal relationships that could have appeared to influence the work reported in this paper.
